# Not all small bowel pneumatosis is ischaemia: a case-based systematic review of non-ischaemic causes

**DOI:** 10.1308/rcsann.2026.0011

**Published:** 2026-03-05

**Authors:** S Olusola, M Choudhry, V Panem, J Wilson, S Rana, C Parmar, M Kathirvel

**Affiliations:** ^1^ Royal Free London NHS Foundation Trust, UK; ^2^ Whittington Health NHS Trust, UK

**Keywords:** Pneumatosis intestinalis, Small bowel, Non-ischaemic, Postoperative ileus, Small bowel obstruction, Systematic review

## Abstract

**Background:**

Pneumatosis intestinalis (PI) involving the small bowel is often interpreted as a radiological surrogate for ischaemia, prompting urgent surgical intervention. However, non-ischaemic causes—particularly in the postoperative setting—are under-recognised. This case-based systematic review aims to explore and synthesise reported non-ischaemic aetiologies of small bowel PI.

**Methods:**

We present the case of a 74-year-old man who developed small bowel PI following ileostomy reversal, with computed tomography findings initially suggestive of bowel ischaemia. In the absence of clinical or biochemical evidence of sepsis, he was managed conservatively and made a full recovery. A systematic review was conducted using PRISMA methodology across PubMed, Embase and Medline databases. Inclusion criteria focused on reports of small bowel PI in the English language. Data were extracted on clinical context, diagnostic modality, treatment and outcomes.

**Results:**

A total of 24 articles were included, comprising one retrospective cohort and 23 case reports, yielding 37 patients. The most frequent aetiology was small bowel obstruction (59.4%). Rarer causes included a rapidly progressive scleroderma-like disease, congenital lymphangioma and mechanical ventilation. Although 72.9% underwent surgical intervention, 27% were successfully managed conservatively. Only 10.8% had a suspected ischaemic component, and six patients died. Radiological overlap with ischaemic PI was common, yet several patients improved without surgery and only two (5.4%) had confirmed transmural small bowel necrosis.

**Conclusions:**

Not all small bowel pneumatosis indicates ischaemia. Conservative management can be effective in carefully selected patients, guided by clinical status and serial imaging. Early recognition of non-ischaemic PI is crucial to avoid unnecessary laparotomy.

## Introduction

Pneumatosis intestinalis (PI), also referred to as pneumatosis cystoides intestinalis, is defined as the presence of gas within the bowel wall and is both a radiological and pathological finding. Although rare, with a reported incidence of 0.03%, the frequency of its detection is increasing—attributable largely to the widespread use of computed tomography (CT) imaging.^1^

PI involving the large intestine is encountered more frequently than small bowel PI and is often associated with benign or non-ischaemic processes. In contrast, pneumatosis affecting the small bowel is relatively rare and typically raises immediate clinical concern due to its strong association with ischaemic bowel. The most common cause of small bowel PI is bowel ischaemia, which may arise from arterial obstruction such as superior mesenteric artery thrombosis or embolism, or venous compromise including portal or mesenteric vein thrombosis. This presumed link between small bowel PI and ischaemia frequently prompts urgent surgical exploration. However, in select clinical contexts, non-ischaemic small bowel PI may occur and, when misdiagnosed, may result in unnecessary operative intervention.

PI is associated classically with bowel ischaemia and is therefore often viewed as an ominous sign. In a recent systematic review, colonic ischaemia accounted for 13.6% of PI cases.^2^ In neonates, its presence is linked closely to acute necrotising enterocolitis. However, while colonic PI is relatively well characterised, non-ischaemic small bowel PI remains uncommon and is discussed less frequently in the literature.

The physiological presence of intraluminal gas is considered normal and is typically expelled via flatulence. In contrast, the detection of gas within the layers of the bowel wall—namely the mucosa, submucosa or subserosa—is considered pathological. Although the exact pathogenesis of PI remains uncertain, three predominant theories have been proposed to explain its occurrence. The mechanical theory suggests that increased intraluminal pressure or mucosal injury (e.g., from obstruction or iatrogenic causes such as surgery or endoscopy) facilitates the translocation of gas into the bowel wall. The bacterial theory proposes that gas-forming organisms, such as *Escherichia coli*, penetrate the mucosa and produce gas within the bowel wall. A related biochemical hypothesis posits that fermentation by luminal bacteria increases hydrogen production and intraluminal pressure, resulting in transmucosal gas diffusion. Finally, the pulmonary theory hypothesises that alveolar rupture from pulmonary conditions (e.g., chronic obstructive pulmonary disease (COPD) or asthma) allows gas to migrate through the mediastinum into the mesenteric vasculature and subsequently into the bowel wall.^3^

PI may be classified as either primary (idiopathic, 15%) or secondary (85%), the latter being more common and usually associated with an underlying pathology.^4^ Clinical presentation is highly variable and depends on the aetiology. While primary or benign PI is often asymptomatic or causes mild abdominal symptoms, secondary PI may present with abdominal pain, vomiting, haematochezia and, in severe cases, features mimicking bowel ischaemia or peritonitis.^5^ Complications such as pneumoperitoneum, perforation and bowel obstruction occur in approximately 3% of cases.^6^

A systematic review of PI revealed abdominal distension and pain as the most common presenting features (23.6%), with haemodynamic instability noted in 14.7% of patients.^2^ Diagnosis is predominantly radiological, with CT imaging being the most sensitive and capable of identifying both the pneumatosis and associated features such as hepatic portal venous gas (PVG).^7^ On imaging, the gas may appear as linear or cystic lucencies depending on whether it resides in the submucosal or subserosal layers. Importantly, both patterns have been observed in both benign and life-threatening cases.^8^ According to Jamart’s histopathological observations, small bowel PI tends to be subserosal, while colonic PI is more frequently submucosal in distribution.^9^

This paper was prompted by a patient managed in our department who developed postoperative small bowel PI, radiologically suspicious for ischaemia, but who ultimately recovered fully with conservative treatment. We aim to highlight the spectrum of non-ischaemic causes of small bowel PI and evaluate clinical outcomes and management approaches through a case-based systematic review.

## Case presentation

A 74-year-old man with a history of midtransverse colon adenocarcinoma invading the greater curvature of the stomach underwent an extended right hemicolectomy with partial gastrectomy and a Roux-en-Y gastrojejunostomy. He completed a course of adjuvant chemotherapy and returned 11 months later for elective ileostomy reversal. His medical history included neuropathic pain and spondylitis.

The patient underwent laparotomy with adhesiolysis, ileostomy takedown and ileocolic anastomosis. His initial postoperative course was complicated by ileus. On postoperative day (POD) three, he developed elevated inflammatory markers including a raised C-reactive protein and leukocytosis. On POD four, localised wound bulging was noted at the ileostomy closure site. An ultrasound confirmed a haematoma, which was subsequently evacuated.

**Figure 1:**
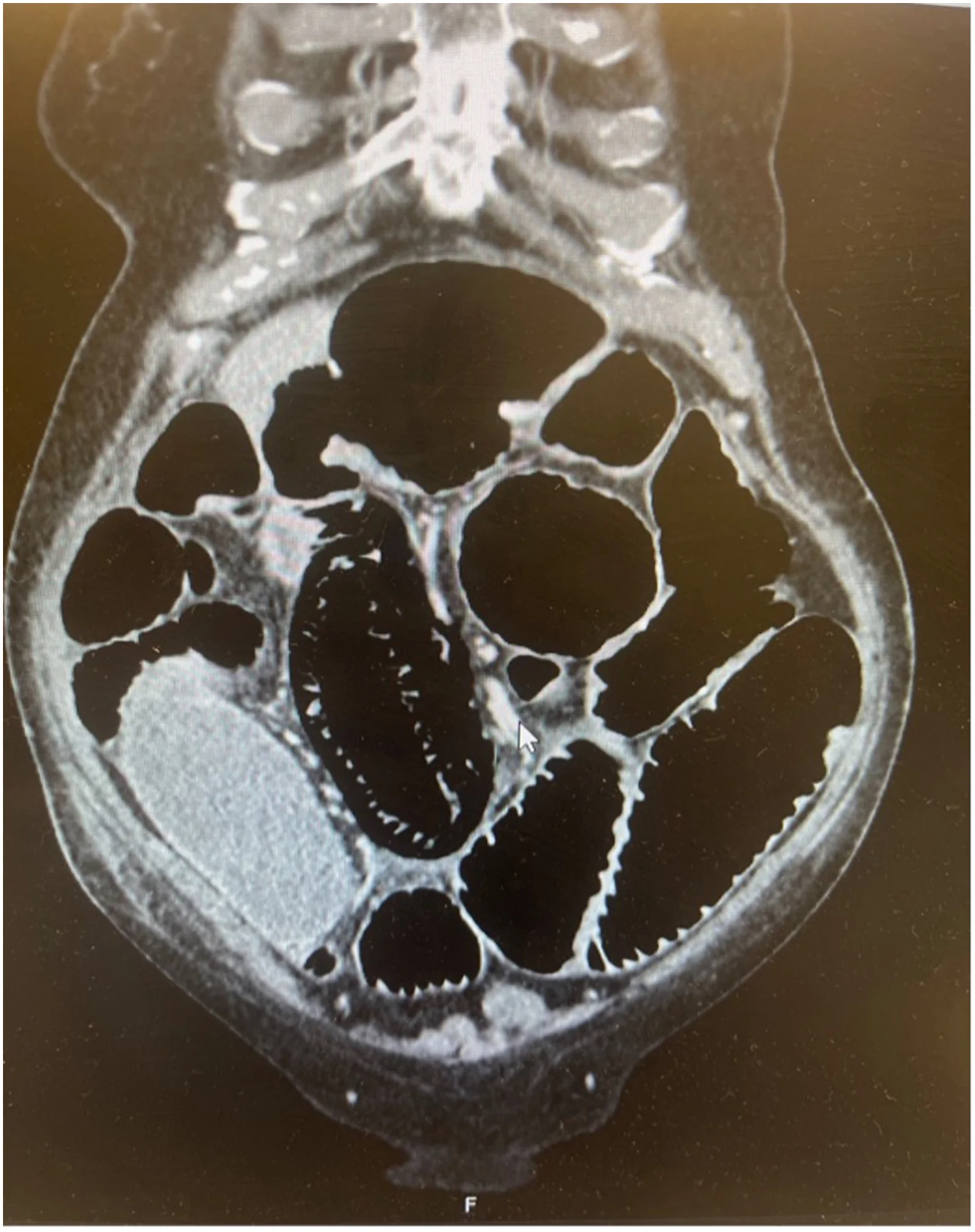
Coronal CT image (POD seven) demonstrating segmental small bowel PI. This coronal section shows multiple loops of dilated small bowel with intramural gas noted along a 20cm segment of the jejunum, distal to the gastrojejunostomy. The pneumatosis appears as small gas collections tracking within the bowel wall, raising concern for possible ischaemia. No pneumoperitoneum or portal venous gas is seen. CT = computed tomography; PI = pneumatosis intestinalis; POD = postoperative day

By POD seven, the patient developed vomiting, abdominal distension and failure to pass flatus or stool. A CT scan demonstrated PI involving a 20cm segment of the jejunum just distal to the gastrojejunostomy, with radiological features raising concern for small bowel ischaemia (Figure 1). Despite these findings, there were no clinical signs of peritonitis or sepsis, and blood lactate levels remained within normal limits.

**Figure 2:**
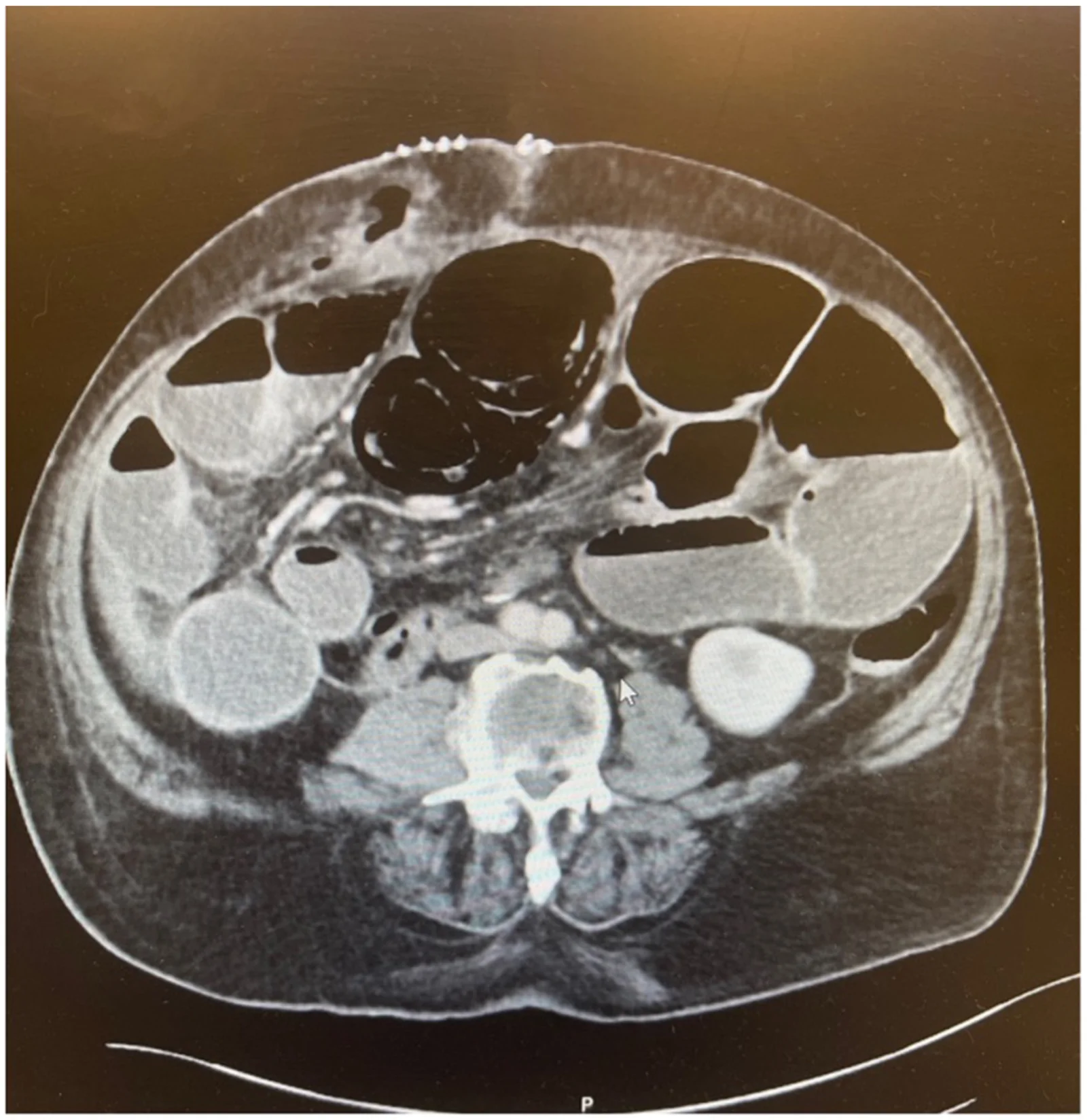
Axial CT image (POD eight) showing improvement in PI following conservative management. Compared with Figure 1, the bowel wall demonstrates preserved enhancement and a marked reduction in intramural gas. There is continued bowel dilation, but no evidence of free intra-abdominal air or portal venous gas. CT = computed tomography; PI = pneumatosis intestinalis; POD = postoperative day

A decision was made to pursue conservative management, including intravenous fluids, antibiotics and close monitoring. Serial lactate measurements were unremarkable. Total parenteral nutrition (TPN) was initiated via a central venous catheter to support nutritional needs. A repeat CT scan on POD eight revealed reduced intramural gas and preserved bowel wall enhancement (Figure 2), supporting a non-ischaemic aetiology.

The patient’s condition improved gradually. By POD nine, inflammatory markers had normalised. Bowel function resumed, and oral intake was progressively advanced. The nasogastric tube was removed on POD12, and the patient was discharged in stable condition on POD15, tolerating a soft diet.

## Methods

A systematic literature review was conducted using the PubMed, Embase and Medline databases. The following search terms were applied:

- ‘Pneumatosis intestinalis’ AND ‘small bowel’;
- ‘Pneumatosis intestinalis’ AND ‘small intestine’;
- ‘Pneumatosis cystoides intestinalis’ AND ‘small bowel’;
- ‘Pneumatosis cystoides intestinalis’ AND ‘small intestine’.

This review was conducted in accordance with the Preferred Reporting Items for Systematic Reviews and Meta-Analyses (PRISMA) guidelines.^10^ Articles were included if they were published in English and described patients with PI involving the small bowel. Studies involving all age groups, genders and ethnicities were considered eligible. Exclusion criteria included non-English articles, unavailability of full text and cases reporting exclusively colonic or non-small bowel PI.

**Figure 3:**
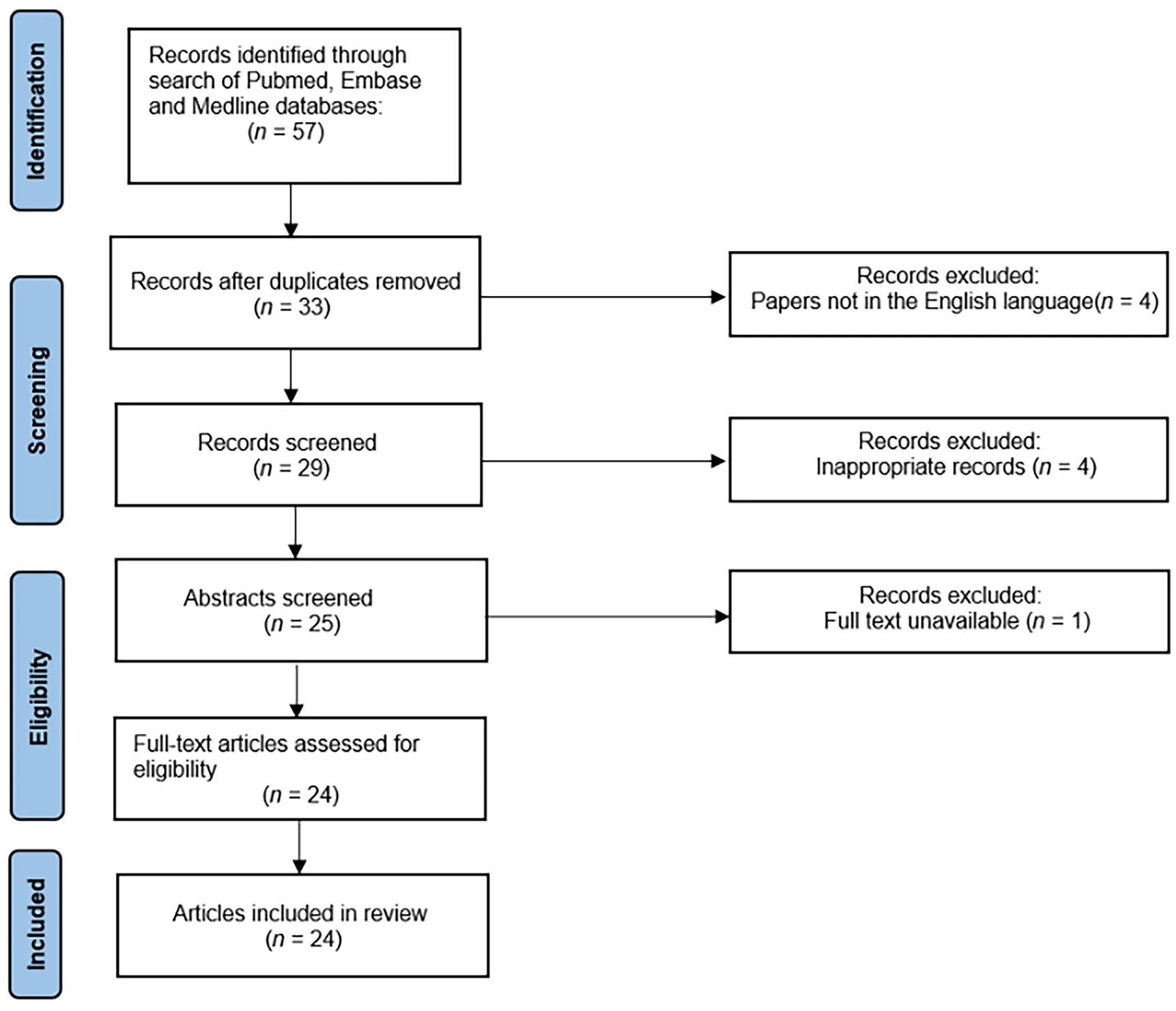
PRISMA flowchart PRISMA = Preferred Reporting Items for Systematic Reviews and Meta-Analyses

Two independent reviewers screened and selected articles based on title, abstract and full-text review. Discrepancies were resolved by consensus. Extracted data included patient demographics, clinical presentation, imaging and laboratory findings, underlying aetiology, complications, management strategies and outcomes. A PRISMA flow diagram is provided in Figure 3.

## Results

The systematic review yielded 57 articles, of which 24 were included after exclusions. This consisted of 23 case reports and one retrospective single-centre cohort study.^11–34^ A total of 37 patients were analysed; their details are summarised in Table 1. Given that the included studies were predominantly case reports, a formal risk-of-bias assessment was not performed.

**Table 1:** Summary of non-ischaemic small bowel pneumatosis intestinalis cases.

Authors	Number of patients	Age	Sex	Clinical presentation	Laboratory results	Diagnostic modality	PI location	Aetiology	Management	Complications	Mortality (Y/N)
Bayissa *et al*^11^	1	25	M	Gastric outlet obstruction	No reported abnormalities	CT abdomen	Ileal	GOO	Surgical: Gastro-jejunal bypass with truncal vagotomy and Braun's anastomosis. Distal ileal resection and ileo-ileo anastomosis	–	N
Zouaghi *et al*^12^	1	60	M	Abdominal pain, distension and vomiting	Raised WCC and CRP	Surgical exploration	Ileal	Small bowel volvulus secondary to an inflammatory appendix	Surgical: detorsion and appendicectomy	Pneumoperitoneum	N
Lebert *et al*^13^	14	59 (mean)	M (7)F (7)	Abdominal pain (71%) nausea/vomiting (100%)	CRP 55 mg/L (mean) lactate 3.1 mmol/L (mean)	CT abdomen (100%)	Jejunal (86%) Ileal (21%)	Mechanical SBO (seven cases were adhesional, three due to external hernia, and one each due to volvulus, peritoneal carcinomatosis, internal hernia and intussusception)	Surgical (71.4%): no bowel resections performed Conservative (28.5%)	All patients had associated PVG	Two mortalities—both from acute cardiopulmonary failure 1 mortality during the post-operative period
Mareth *et al*^14^	1	42	F	Decreased ileostomy output with nausea and vomiting on the background of partial SBO	Not reported	AXR and CT abdomen	Not specified	SBO	Conservative: bowel rest and NG decompression	–	N
Ng *et al*^15^	1	70	M	Abdominal pain, distention and absolute constipation	Lactate 3.3 mmol/L	CT abdomen and pelvis	Distal oesophagus, stomach and small bowel	Bowel ischaemia	Surgical: resection of ischaemic small bowel	Extensive portomesenteric venous gas Postoperative intrabdominal abscess and portal vein thrombosis	N
Marley *et al*^16^	1	88	M	Abdominal pain and vomiting	Not reported	CT abdomen	Not specified	Partial SBO: small bowel volvulus secondary to adhesions	Surgical: volvulus secondary to adhesions was divided and reduced. No small bowel resection	Free intraperitoneal air	N
Slesser *et al*^17^	1	79	M	Chronic abdominal distention, pain and vomiting	Normocytic anaemia	CT abdomen and pelvis	From duodenal-jejunal flexure to terminal ileum	Small bowel volvulus secondary to a long and hypermobile small bowel mesentery	Underwent elective surgical exploration- no resection performed	Free intraperitoneal air	N
Alkhatib *et al*^18^	1	60	M	Abdominal pain with nausea and vomiting	AKI with a leucocytosis	CT abdomen	Not specified	SBO secondary to internal hernia or adhesions	NA	PVG present	Y—patient developed severe hypoxaemia, bradycardia and hypotension before surgical intervention
Arora *et al*^19^	1	27	M	Acute abdomen	Not reported	AXR and visualised during emergency laparotomy. CT abdomen not done due to the emergency nature of the presentation	Terminal small bowel	Perforated duodenal ulcer leading to extravasation of its content including air causing dissection along mesenteric vessels	Surgical: patch omentoplasty, limited right colectomy and resection of the involved small bowel	Pneumoperitoneum	N
Liau *et al*^20^	1	76	M	Chronic altered bowel habit, abdominal pain and distention Re-presented 6 weeks later with sudden onset abdominal pain, peritonitis, distention and vomiting	First presentation: unremarkable Second presentation: raised WCC and significant metabolic acidosis	CT abdomen	Ileum	Intermittent reversible ischaemia secondary to intermittent and reversible volvulus	Surgical: extensive resection of infarcted small bowel	Benign pneumoperitoneum seen on first presentation Extensive small bowel infarction	Y—persistent postoperative hypotension
Shimanuki *et al*^21^	1	70	M	Abdominal pain, bloating and altered bowel habit	Unremarkable	AXR and CT abdomen	Entire small bowel	Presumed bowel ischaemia	Surgical: small bowel resection	–	N
Smith *et al*^22^	1	38	F	Postoperative abdominal pain and delirium	Raised WCC and urea	AXR	Not specified	Necrotising enteritis occurring as a post-operative complication	Conservative: IV antibiotics and IV fluids	–	N
Kim *et al*^23^	1	70	M	Sudden onset abdominal pain and distention while on mechanical ventilation for aspiration pneumonia	Raised WCC and a lactate of 2.5 mmol/L	AXR and CT abdomen	Ileal	The use of mechanical ventilation in a patient with emphysema	Surgical: small bowel resection	SBO with mesenteric torsion and volvulus	N
Berrito *et al*^24^	1	86	M	First presentation: abdominal pain. Second presentation: re-presented a month later with generalised abdominal pain	Unremarkable	CT abdomen	Jejunum and ileum	Unclear aetiology	Surgical: small bowel resection and adhesion lysis	Free intraperitoneal air	N
Light *et al*^25^	1	67	F	Abdominal pain	Unremarkable	CT abdomen	Small bowel with some extension to the caecum and ascending colon	Acute intestinal ischaemia secondary to small bowel volvulus	Surgical: small bowel resection	–	Y—Postoperative respiratory failure
Anne *et al*^26^	1	63	M	First presentation: abdominal pain with nausea and vomiting. Second presentation: represented 2 months later with similar symptoms	Unremarkable	CT abdomen	Not specified	Unclear aetiology	Conservative: first presentation: bowel rest and antibiotics Second presentation: TPN and bowel rest for 1 month. Repeat CT demonstrated resolution of the PI	–	N
Williams *et al*^27^	1	42	F	Change in bowel habit with weight loss	Unremarkable	Surgical exploration with laparotomy	Distal ileum extending to the transverse colon	Unclear aetiology	Surgical: lymph node removal only. No small bowel resection	–	N
Chaves *et al*^28^	1	85	F	Abdominal pain and distention with nausea and vomiting	Metabolic acidosis	Surgical exploration with emergency laparotomy	Not specified	Unclear aetiology	Surgical: small bowel resection with anastomosis	Small bowel volvulus, internal hernia with signs of intestinal ischaemia. Pneumoperitoneum	N
Strahm *et al*^29^	1	71	M	Abdominal pain Patient known to have had PI three years before acute presentation and had intermittent self-limiting symptoms during this period	Not reported	PI originally visualised using CT and abdominal MRI	Small bowel	Unclear aetiology	Surgical: small bowel resection	Mesenteric torsion and SBO Free intraperitoneal gas	N
Aboubakr *et al*^30^	1	61	F	Chronic abdominal distention with change in bowel habit	Unremarkable	CT abdomen	Not specified	Rapidly progressive systemic scleroderma-like disease	Conservative: bowel rest, steroids and rituximab. Repeat CT two months later showed resolution of the PI	–	N
Harris *et al*^31^	1	57	F	Abdominal pain with nausea and vomiting	Unremarkable	Surgical exploration with emergency laparotomy	Jejunum	Small bowel lymphangioma	Surgical: jejunal resection	Free air in the peritoneum	N
Rathi *et al*^32^	1	47	M	Chronic abdominal pain with distention and vomiting	Unremarkable	CT abdomen	Ileum	Intermittent and recurrent twisting of hypermobile mesentery likely resulting in ischaemia	Surgical: ileal resection even though there was no evidence of bowel ischaemia	Pneumoperitoneum	N
Seto *et al*^33^	1	50	F	Abdominal fullness with nausea and vomiting	Unremarkable	CT abdomen	Jejunum	SBO secondary to adenocarcinoma	Surgical: jejunal resection	Mesenteric gas	N
Paw *et al*^34^	1	72	M	First presentation: chronic abdominal pain with change in bowel habit and weight loss. Second presentation: Readmitted two weeks later with similar symptoms	Not mentioned	Barium meal and follow through	Not specified	Unknown aetiology	Conservative: IV fluids, TPN and hyperbaric oxygen therapy	–	N

AKI = acute kidney injury; AXR = abdominal X-ray; CRP = C-reactive protein; CT = computed tomography; F = female; GOO = gastric outlet obstruction; IV = intravenous; M = male; MRI = magnetic resonance imaging; N = no; NA = not applicable; NG = nasogastric; PI = pneumatosis intestinalis; PVG = portal venous gas; SBO = small bowel obstruction; TPN = total parenteral nutrition; WCC = white cell count; Y = yes

The mean age was 60.3 years (range: 25–93), with a male predominance of 59.4% (*n*=22). Common presenting symptoms included abdominal pain, nausea, vomiting and altered bowel habits. Raised white cell count and elevated lactate were the most frequent laboratory abnormalities. Diagnosis was confirmed by CT in most cases, with another five identified intraoperatively and two by other imaging modalities.

### Aetiologies

- Small bowel obstruction (SBO) was the most common cause (22 patients, 59.4%), with underlying mechanisms including adhesions, volvulus, hernias, peritoneal carcinomatosis, intussusception and jejunal tumours.
- Gastric outlet obstruction and a perforated duodenal ulcer were reported in two patients.^11,19^
- Necrotising enteritis as a postoperative complication was reported in one patient.^22^
- Rarer causes included systemic sclerosis,^30^ congenital lymphangioma and mechanical ventilation —each reported in a single patient.^23,31^
- In six patients (16.2%), no clear cause was identified.
- Only 10.8% had a suspected ischaemic component.^15,20,21,25^
- In three cases, PI extended beyond the small bowel.^15,25,27^

### Ischaemia and surgical management

Only two patients (from Ng *et al* and Liau *et al*) had confirmed transmural small bowel necrosis, resulting in one death.^15,20^ Three others underwent resection for suspected non-viability, but without documented histological evidence of necrosis.^11,21,24^ This highlights the challenge in distinguishing non-ischaemic from ischaemic PI based solely on imaging.

PVG was noted in 16 patients, 14 of whom were part of the retrospective study by Lebert *et al*,^13^ where PVG was an inclusion criterion. Two other patients had PVG detected incidentally.^15,18^

Surgical intervention was performed in 27 patients (72.9%). Of these, 13 underwent small bowel resection, due primarily to clinical suspicion of non-viable bowel. In some cases, additional procedures were performed:

- Gastrojejunostomy and adhesiolysis for inflammatory distortion (Bayissa *et al*)^11^
- Appendicectomy for appendicitis-associated volvulus (Zouaghi *et al*)^12^
- Patch omentoplasty following a perforated duodenal ulcer (Arora *et al*).^19^

Conservative management was used in nine patients (24.3%), including bowel rest, intravenous antibiotics, total parenteral nutrition and, in one case, hyperbaric oxygen therapy.^34^ All patients managed non-operatively improved on follow-up; however, five patients initially treated conservatively re-presented with clinical deterioration.^20,24,26,29,34^ Of these five patients:

- One died of acute decline before surgical intervention^20^;
- Two required delayed surgery^24,29^;
- Two recovered with further conservative treatment.^26,34^

In four of these five patients, the aetiology was not clearly defined in the original reports.

In total, six deaths were recorded. Aside from the two patients with confirmed bowel necrosis,^15,20^ causes were either multifactorial or not documented explicitly.

## Discussion

The first reported occurrence of PI was by DuVernoi during cadaveric dissection in 1730.^35^ Since then, cases involving both the colon and small bowel have been reported across a wide clinical spectrum. The various aetiologies of PI can be grouped into distinct pathophysiological categories, as summarised in Table 2.^36^ These aetiologies can typically be explained by one or more of the mechanical, bacterial, biochemical or pulmonary hypotheses discussed earlier.

**Table 2:** Reported causes of PI^36^

Infectious	*Clostridium difficile*, HIV, AIDS, cytomegalovirus
Inflammatory	Crohn's disease, ulcerative colitis, autoimmune conditions
Pulmonary disease	COPD, asthma, cystic fibrosis
Iatrogenic	Endoscopy, postsurgical anastomosis, mechanical ventilation
Drug-induced	Corticosteroids, anti-cancer therapies
Gastrointestinal pathology	Bowel obstruction, bowel ischaemia, volvulus

AIDS = acquired immunodeficiency syndrome; COPD = chronic obstructive pulmonary disease; HIV = human immunodeficiency virus; PI = pneumatosis intestinalis

From our systematic review, the most common cause of non-ischaemic small bowel PI was SBO, reported in 22 of 37 patients. In such cases, increased intraluminal pressure likely leads to mucosal injury and gas dissection into the bowel wall, consistent with the mechanical theory. Additionally, epithelial damage can increase permeability as early as three hours after an obstruction begins,^37^ while stagnation of contents may facilitate gas-forming bacterial overgrowth, aligning with the bacterial theory.^38^

Interestingly, PI itself may become a secondary contributor to SBO. In several cases, large or numerous cysts were hypothesised to narrow the bowel lumen, leading to mechanical occlusion.^39^ In Berritto *et al*, cyst rupture led to inflammatory adhesions requiring operative management, supporting this theory.^24^

Our review identified rare, non-ischaemic causes in single-patient reports:

- Mechanical ventilation, in a patient with emphysema, likely contributed to alveolar rupture and gas dissection along vascular sheaths into the bowel wall.^23^
- Congenital lymphangioma was associated with lymphatic dysfunction as a proposed mechanism.^31^
- A rapidly progressive systemic scleroderma-like disease was postulated to induce PI via impaired motility and bacterial overgrowth.^30^

In our own case, we propose that PI was a postoperative phenomenon, resulting from mucosal disruption due to bowel handling, compounded by paralytic ileus. The reduction in motility may have increased intraluminal pressure, allowing gas entry into the bowel wall. No features of ischaemia were present clinically or biochemically, and the patient responded to conservative treatment.

At present, no standardised algorithm exists for the management of PI. Management is determined fundamentally by clinical context. The most concerning aetiology is bowel ischaemia, particularly in the postoperative period, and it must be actively ruled out.^40^ This requires integration of physical findings (e.g., peritonitis), biochemical markers (e.g., lactate, acidosis) and haemodynamic stability.

Importantly, radiological features alone are not sufficient to determine the need for surgery. For instance, the presence of pneumoperitoneum may result from benign cyst rupture, rather than true perforation. Similarly, PVG, long thought to be an ominous sign with mortality rates up to 37%,^41^ was present in 16 patients in our review—yet 14 of those were from a single retrospective study where PVG was an inclusion criterion.^13^ Of the two additional cases with PVG (Ng *et al* and Alkhatib *et al*),^15,18^ one was managed with bowel resection and the other died before surgical intervention. Thus, as Perrone *et al* also reported,^2^ PVG should not be considered a sole indication for surgery.

Surgical resection in our cohort was often performed in the absence of histologically confirmed necrosis. In total, 13 patients underwent small bowel resection. Only two patients (in Liau *et al* and Ng *et al*) had documented transmural necrosis.^15,20^ Others were resected due to suspected non-viability or to prevent future episodes:

- Berritto *et al* performed resection due to severe adhesions from asymptomatic cyst rupture.^24^
- Bayissa *et al* resected for inflammatory distortion of the ileum.^11^
- Shikumaki *et al* resected due to dilated and distended small bowel in the presence of bloody fluid.^21^

These findings highlight a key challenge: while prompt resection is mandatory in the presence of confirmed ischaemia, several resections in this series were precautionary rather than definitive and many patients improved without surgery.

Conservative management was used successfully in nine patients (27%), incorporating bowel rest, intravenous fluids, antibiotics, parenteral nutrition and, in some cases, hyperbaric oxygen therapy.^34^ Metronidazole was used in multiple reports as an antimicrobial targeting gas-producing anaerobes.^42^ Hyperbaric oxygen may also assist by both suppressing anaerobic flora and facilitating gas resorption.^43^

Among five patients who initially received conservative management, clinical deterioration prompted re-evaluation:

- One patient died before surgery due to acute deterioration.^20^
- Two underwent delayed surgical intervention.^24,29^
- Two were again managed conservatively and improved.^26,34^

These outcomes underscore the need for close monitoring, even in patients initially deemed stable.

When managing patients with small bowel PI, we believe surgical exploration is warranted in the presence of the following features:

– Clinical or haemodynamic instability;
– Concerning clinical examination (peritonism);
– Biochemical derangement, including elevated lactate or metabolic acidosis;
– The presence of an underlying aetiology requiring operative management.

In the absence of these factors, a conservative approach with close clinical observation, serial imaging and lactates is appropriate.

## Limitations

Our review is limited by the inherent nature of case reports and single-centre data, which introduces selection and publication bias. Furthermore, 16.2% of included cases had no identified aetiology, and comorbidity data were reported inconsistently. Despite these limitations, this case-based synthesis offers important insight into non-ischaemic causes of small bowel PI.

## Conclusions

The longstanding association between PI and bowel ischaemia has led many clinicians to consider it a surgical emergency. However, our systematic review highlights that small bowel PI can arise from a variety of non-ischaemic causes—most commonly SBO, but also rare entities such as connective tissue disorders, lymphatic anomalies and postoperative ileus.

Surgeons should adopt a nuanced, case-by-case approach when encountering small bowel PI. Over-reliance on CT findings risks unnecessary laparotomy, while under-recognition of ischaemia may delay life-saving intervention. This review advocates for clinical vigilance, serial monitoring and assessment, multidisciplinary decision-making and greater awareness of non-ischaemic mimics in the management of small bowel PI.

## Funding

The author/s received no financial support for the research, authorship and/or publication of this article.

## Competing interests

The author/s declare no competing interests.

## Patient consent

Signed patient consent has been obtained.

## Ethics approval statement

The authors are accountable for all aspects of the work in ensuring that questions related to the accuracy or integrity of any part of the work are appropriately investigated and resolved. Written informed consent was obtained from the patient for publication. Board institutional approval was not required.

## Author contributions

**S Olusola:** Conceptualisation, Data curation, Formal analysis, Investigation, Methodology, Resources, Software, Validation, Visualisation, Writing – original draft, Writing – review & editing. **M Choudhry:** Conceptualisation, Data curation, Formal analysis, Investigation, Methodology, Supervision, Visualisation, Writing – original draft, Writing – review & editing. **V Panem:** Conceptualisation, Formal analysis, Investigation, Methodology, Writing – original draft, Writing – review & editing. **J Wilson:** Conceptualisation, Formal analysis, Methodology, Supervision, Validation, Writing – review & editing. **S Rana:** Conceptualisation, Formal analysis, Supervision, Writing – review & editing. **C Parmar:** Conceptualisation, Formal analysis, Methodology, Supervision, Validation, Visualisation, Writing – review & editing. **M Kathirvel:** Conceptualisation, Data curation, Formal analysis, Investigation, Methodology, Project administration, Supervision, Validation, Visualisation, Writing – original draft, Writing – review & editing.

## Artificial Intelligence

The authors declare that no AI was used to conduct the study or prepare the manuscript.

## References

[1] Kang G. Benign pneumatosis intestinalis: dilemma for primary care clinicians. *Can Fam Physician* 2017; **63**: 766–768. 29025802 PMC5638473

[2] Perrone G, Giuffrida M, Donato V *et al.* The challenge of pneumatosis intestinalis: a contemporary systematic review. *J Pers Med* 2024; **14**: 167. 38392601 10.3390/jpm14020167 PMC10890206

[3] Khalil PN, Huber-Wagner S, Ladurner R *et al.* Natural history, clinical pattern, and surgical considerations of pneumatosis intestinalis. *Eur J Med Res* 2009; **14**: 231–239. 19541582 10.1186/2047-783X-14-6-231 PMC3352014

[4] Heng Y, Schuffler MD, Haggitt RC *et al.* Pneumatosis intestinalis: a review. *Am J Gastroenterol* 1995; **90**: 1747–1758. 7572888

[5] Wu LL, Yang YS, Dou Y *et al.* A systemic analysis of pneumatosis cystoids intestinalis. *World J Gastroenterol* 2013; **19**: 4973–4978. 23946603 10.3748/wjg.v19.i30.4973 PMC3740428

[6] Ling F, Guo D, Zhu L. Pneumatosis cystoides intestinalis: a case report and literature review. *BMC Gastroenterol* 2019; **19**: 176. 31694581 10.1186/s12876-019-1087-9 PMC6836417

[7] Azzaroli F, Turco L, Ceroni L *et al.* Pneumatosis cystoides intestinalis. *World J Gastroenterol* 2011; **17**: 4932–4936. 22171137 10.3748/wjg.v17.i44.4932 PMC3235639

[8] Ho LM, Paulson EK, Thompson WM. Pneumatosis intestinalis in the adult: benign to life-threatening causes. *AJR Am J Roentgenol* 2007; **188**: 1604–1613. 17515383 10.2214/AJR.06.1309

[9] Jamart J. Pneumatosis cystoides intestinalis. A statistical study of 919 cases. *Acta Hepatogastroenterol (Stuttg)* 1979; **26**: 419–422. 525221

[10] Page MJ, McKenzie JE, Bossuyt PM *et al.* The PRISMA 2020 statement: an updated guideline for reporting systematic reviews. *BMJ* 2021; **372**: n71. 33782057 10.1136/bmj.n71 PMC8005924

[11] Bayissa BB, Fanos B, Tufa DW *et al.* Extensive inflammatory adhesion of small bowel with massive pneumatosis intestinalis in a patient with gastric outlet obstruction: a rare case report. *Int J Surg Case Rep* 2024; **122**: 110152. 39154563 10.1016/j.ijscr.2024.110152 PMC11378174

[12] Zouaghi A, Hadded D, Meryam M *et al.* Case report: an unusual case of small bowel volvulus due to appendicitis associated with pneumatosis intestinalis: review of the literature. *F1000Res* 2021; **10**: 951. 36483602 10.12688/f1000research.73042.1 PMC9706145

[13] Lebert P, Ernst O, Zins M *et al.* Pneumatosis intestinalis and portal venous gas in mechanical small bowel obstruction: is it worrisome? *Diagn Interv Imaging* 2021; **102**: 545–551. 34030989 10.1016/j.diii.2021.05.001

[14] Mareth K, Alsaad AA, Roy A. Pneumatosis intestinalis in small bowel obstruction. *BMJ Case Rep* 2018; **2018**: bcr2018224684. 10.1136/bcr-2018-224684 PMC5878243 29574437

[15] Ng DW, Chia CS, Tan GH *et al.* A case of portomesenteric venous gas found with extensive secondary pneumatosis intestinalis in the small bowel, stomach and distal oesophagus on radiographic images with an insidious presentation. *Clin J Gastroenterol* 2014; **7**: 140–143. 26183630 10.1007/s12328-014-0464-8

[16] Marley WD, Dodd B, Spence G. Pneumatosis intestinalis presenting as a partial small bowel obstruction. *BMJ Case Rep* 2012; **2012**: bcr2012007291. 10.1136/bcr-2012-007291 PMC4543548 23257270

[17] Slesser AA, Patel PH, Das SC *et al.* A rare case of segmental small bowel pneumatosis intestinalis: a case report. *Int J Surg Case Rep* 2011; **2**: 185–187. 22096722 10.1016/j.ijscr.2011.06.003 PMC3199630

[18] Alkhatib AA, Elkhatib FA, Alkhatib OF *et al.* Pneumatosis intestinalis and gas in portal vein associated with small bowel obstruction. *J Emerg Med* 2011; **40**: e125–e126. 19327927 10.1016/j.jemermed.2009.02.008

[19] Arora R, El-Hameed AA, Al Harbi O. Pneumatosis intestinalis of small bowel in an adult: a case report. *Kuwait Med J* 2009; **41**: 143–145.

[20] Liau SS, Cope C, MacFarlane M *et al.* A lethal case of pneumatosis intestinalis complicated by small bowel volvulus. *Clin J Gastroenterol* 2009; **2**: 22–26. 26191803 10.1007/s12328-008-0038-8

[21] Shimanuki K, Nomura T, Hiramoto Y *et al.* Pneumatosis intestinalis in the small bowel of an adult: report of a case. *Surg Today* 2001; **31**: 246–249. 11318130 10.1007/s005950170178

[22] Smith EEJ, Saunders JH, Bowley N. Pneumatosis intestinalis in the small bowel of an adult—a radiological sign of a serious post-operative complication. *Br J Radiol* 1981; **54**: 266–267. 7470794 10.1259/0007-1285-54-639-266

[23] Kim DJ, Choi YJ, Yoo YS. Pneumatosis intestinalis presenting as small bowel obstruction without bowel ischemia after mechanical ventilation. *Acute Crit Care* 2019; **34**: 81–85. 31723909 10.4266/acc.2016.00311 PMC6849051

[24] Berritto D, Crincoli R, Iacobellis F *et al.* Primary pneumatosis intestinalis of small bowel: a case of a rare disease. *Case Rep Surg* 2014; **2014**: 350312. 25478280 10.1155/2014/350312 PMC4248370

[25] Light D, Robinson A, Hennessy C. Pneumatosis intestinalis of the small bowel; radiological and intra-operative findings. *J Surg Case Rep* 2010; **2010**: 3. 10.1093/jscr/2010.3.3 PMC3649088 24946173

[26] Anne N, Rajput A, Dunn KB *et al.* Idiopathic pneumatosis intestinalis of the small intestine. *Am Surg* 2008; **74**: 1127–1129. 19062680

[27] Williams MJ, Sutherland DH, Clark CG. Lymphosarcoma of the small intestine with a malabsorption syndrome and pneumatosis intestinalis. Report of a case with peroral jejunal biopsy. *Gastroenterology* 1963; **45**: 550–557. 14070429

[28] Chaves CE R, Fonseca JF, Ballen N *et al.* An unusual case of small bowel volvulus due to pneumatosis cystoides intestinalis: case report and literature review. *Int J Surg Case Rep* 2024; **116**: 109328. 38320416 10.1016/j.ijscr.2024.109328 PMC10850951

[29] Strahm R, Pratsinis A, Jochum AK *et al.* Idiopathic Pneumatosis cystoides intestinalis of the small bowel with pneumoperitoneum and consecutive small bowel mesenteric torsion. A case report. *Int J Surg Case Rep* 2024; **115**: 109220. 38194864 10.1016/j.ijscr.2024.109220 PMC10819719

[30] Aboubakr A, Ghosh G, Wan D. Pneumatosis cystoides intestinalis and small bowel dysmotility in a patient with a scleroderma-like connective tissue disease. *Am J Gastroenterol* 2020; **115**: S1446.

[31] Harris E, Giannotti G. Is pneumatosis cystoides intestinalis a lymphatic pathology?: a case of small bowel lymphangioma and subsequent development of pneumatosis cystoides intestinalis in a 57-year-old female. *J Surg Case Rep* 2019; **2019**: rjy334. 30697407 10.1093/jscr/rjy334 PMC6344922

[32] Rathi C, Pipaliya N, Poddar P *et al.* A rare case of hypermobile mesentery with segmental small bowel pneumatosis cystoides intestinalis. *Intest Res* 2015; **13**: 346–349. 26576141 10.5217/ir.2015.13.4.346 PMC4641862

[33] Seto T, Koide M, Taniuchi N *et al.* Pneumatosis cystoides intestinalis complicating carcinoma of the small intestine. *Am J Surg* 2001; **182**: 287–288. 11587694 10.1016/s0002-9610(01)00710-3

[34] Paw HG, Reed PN. Pneumatosis cystoides intestinalis confined to the small intestine treated with hyperbaric oxygen. *Undersea Hyperb Med* 1996; **23**: 115–117. 8840480

[35] Koss LG. Abdominal gas cysts (pneumatosis cystoides intestinorum hominis); an analysis with a report of a case and a critical review of the literature. *AMA Arch Pathol* 1952; **53**: 523–549. 14923068

[36] Donovan S, Cernigliaro J, Dawson N. Pneumatosis intestinalis: a case report and approach to management. *Case Rep Med* 2011; **2011**: 571387. 21331331 10.1155/2011/571387 PMC3038658

[37] Hartmann L, Zhao X, Macheroux T *et al.* Time-dependent alterations of gut wall integrity in small bowel obstruction in mice. *J Surg Res* 2019; **233**: 249–255. 30502255 10.1016/j.jss.2018.07.038

[38] Bures J, Cyrany J, Kohoutova D *et al.* Small intestinal bacterial overgrowth syndrome. *World J Gastroenterol* 2010; **16**: 2978–2990. 20572300 10.3748/wjg.v16.i24.2978 PMC2890937

[39] Rachid MG, Sadok T, Narjis Y *et al.* Occlusive syndrome in intestinal cystic pneumatosis, medical treatment or surgery. *PAMJ Clin Med* 2020; **2**: 65.

[40] Higashizono K, Yano H, Miyake O *et al.* Postoperative pneumatosis intestinalis (PI) and portal venous gas (PVG) may indicate bowel necrosis: a 52-case study. *BMC Surg* 2016; **16**: 42. 27391125 10.1186/s12893-016-0158-x PMC4938969

[41] Knechtle SJ, Davidoff AM, Rice RP. Pneumatosis intestinalis. Surgical management and clinical outcome. *Ann Surg* 1990; **212**: 160–165. 2375647 10.1097/00000658-199008000-00008 PMC1358051

[42] Tak PP, Van Duinen CM, Bun P *et al.* Pneumatosis cystoides intestinalis in intestinal pseudoobstruction: resolution after therapy with metronidazole. *Dig Dis Sci* 1992; **37**: 949. 1587203 10.1007/BF01300397

[43] Tahiri M, Levy J, Alzaid S *et al.* An approach to pneumatosis intestinalis: factors affecting your management. *Int J Surg Case Rep* 2015; **6C**: 133–137. 25531306 10.1016/j.ijscr.2014.12.007 PMC4334205

